# Drug Coated Balloon in the Treatment of De Novo Coronary Artery Disease: A Narrative Review

**DOI:** 10.3390/jcm12113662

**Published:** 2023-05-25

**Authors:** Filippo Zilio, Monica Verdoia, Maria Carmen De Angelis, Federico Zucchelli, Marco Borghesi, Andrea Rognoni, Roberto Bonmassari

**Affiliations:** 1 Department of Cardiology, Santa Chiara Hospital, 38122 Trento, Italy; 2 Nuovo Ospedale degli Infermi, 13900 Biella, Italy; 3 U.O. Cardiologia-UTIC-Emodinamica, Ospedale del Mare, 80147 Napoli, Italy

**Keywords:** drug coated balloon, percutaneous coronary artery intervention, paclitaxel, sirolimus

## Abstract

Drug coated balloons (DCBs) are currently indicated in guidelines as a first choice option in the management of instant restenosis, whereas their use in de novo lesions is still debated. The concerns raised after the contrasting results of the initial trials with DCBs in de novo lesions have been more recently overcome by a larger amount of data confirming their safety and effectiveness as compared to drug-eluting stents (DES), with potentially greater benefits being achieved, especially in particular anatomical settings, as in very small or large vessels and bifurcations, but also in selected subsets of higher-risk patients, where a ‘leave nothing behind’ strategy could offer a reduction of the inflammatory stimulus and thrombotic risk. The present review aims at providing an overview of current available DCB devices and their indications of use based on the results of data achieved so far.

## 1. Introduction

Drug coated balloons (DCBs) appeared in the European market of interventional cardiology in 2007, with the aim of offering a combined therapeutic, mechanical (linked to balloon dilatation) and biological (ensured by drug release in the vessel wall) solution, furthermore, avoiding the implantation of a permanent prosthesis [1].

Nowadays, drug-eluting stents (DES) are considered the treatment of choice for percutaneous coronary revascularization but their use still presents certain limitations, including in particular anatomical settings, such as small vessels or bifurcations, and these limitations relate to clinical conditions, such as increased bleeding or thrombotic risk [2]. To overcome some of these limitations, DCBs have been developed in recent years [1,3]. DCBs are balloons with a variable degree of compliance, coated with an antiproliferative drug that is rapidly released upon contact with the wall. DCBs are designed to deliver an antiproliferative drug and not to treat the stenosis. Therefore, before their use, the lesion must be adequately pre-treated and the device is then inflated for a long time (30–120 s), which allows an adequate transfer of the drug to the vessel wall [3].

DCBs offer some theoretical advantages over DES. One of the advantages of DCBs compared to DES is that they provide a larger contact surface with the vessel, allowing a more homogeneous drug–tissue transfer. Moreover, the lack of a permanent prosthesis in the vessel favors the restoration of regular vasomotion and the possibility of reducing the duration of the dual antiplatelet therapy. In this way, the mechanical expansion of the vessel is combined with the release of an antiproliferative drug, which begins its journey inside the vascular wall from the intima to the media and adventitia: in these last two locations, in fact, the drug will promote a physiological healing of the vessel with a positive remodeling and potential lumen gain [4].

In addition to the ‘local’ benefits for the treated lesion, different trials and meta-analyses [5,6,7,8] have underlined a trend for better clinical outcomes and reduced all-cause mortality with DCBs as compared to DES, although these findings and the exact pathophysiological basis for this observation still deserve further investigation [4].

The aim of this review is to provide an overview on the available data and current DCB devices, focusing on de novo lesions and particular anatomical or patient subsets.

## 2. DCBs Characteristics according to Antiproliferative Drugs

### 2.1. Paclitaxel-Coated Balloons

The most widely studied drug in the setting of DCBs is paclitaxel, whose physicochemical properties seem to make the substance most suitable for this application [9]. Different paclitaxel formulations have been used, including drug-only coatings as well as combinations with small fractions (typically 10%) of different additives, such as iopromide, urea, butyryl trihexyl citrate or a combination of polysorbate and sorbitol. Paclitaxel is a lipophilic drug that rapidly crosses the cell membrane of smooth muscle cells and binds to microtubules, stabilizing them during mitosis, thus inhibiting cell division and migration, and therefore, cell proliferation [10]. The dosage range is between 2 and 3.5 μg/mm^2^ of inflated balloon surface. The coating (matrix or carrier) of the balloon is essential because it must be able to retain the drug during the transit of the lesion and ensure a rapid and homogeneous transfer to the vessel wall during inflation, reducing the risk of dispersion. Paclitaxel is typically applied into the balloon surface at a concentration of 3 mg/mm^2^. Each type of paclitaxel-coated balloon (pDCB) is characterized by a different drug/excipient system, because if paclitaxel is applied as a firm compound, the required bioavailability is not obtained, as demonstrated in studies on porcine coronary overstretch models [11]. DCBs coated with paclitaxel in a water-soluble matrix have shown beneficial effects in the treatment and prevention of restenosis in the porcine and in humans, for both coronary in-stent restenosis and in peripheral arteries [12,13]. DCBs based on the Paccocath^®^ technology (SeQuent Please) is widely available: in this case, the balloon is coated with a homogenous matrix of paclitaxel and contrast media (iopromide). This last drug acts as a ‘spacer’ and, thereby, makes the coating porous and paclitaxel bioavailable. Therefore, the matrix allows a reliable release and enables an immediate uptake into the vascular wall of paclitaxel. The hydrophilic character of iopromide and the lipophilic properties of paclitaxel support the release of the drug from the balloon surface and its delivery into the vascular wall. The Paccocath^®^ technology has long term efficacy with a short term exposure: after a ‘single shot’ application of paclitaxel, there is a sustained antiproliferative action on smooth muscle cells over 14 days in absence of cytotoxic effects. Following such single drug delivery, the paclitaxel concentration reaches bottom levels in vascular cells after 24 h [14]. However, other additives or strategies to release the antiproliferative drug have been tested; for example, the Dior balloon has a ‘nanoporous’ balloon surface containing paclitaxel microcrystals following dimethyl sulfoxide treatment [15]. Commercially available DCBs’ characteristics are summarized in Table 1 together with the references of the most important studies [6,16,17,18,19,20,21,22,23,24,25,26,27,28,29,30,31,32,33,34,35,36,37,38,39,40,41,42,43,44,45,46,47,48,49,50,51,52,53,54,55,56].

### 2.2. Sirolimus-Coated Balloons

Although paclitaxel presents the most robust data for PTCA balloon coating, ‘limus’-eluting stents are currently dominating the scenario of coronary interventions for drug eluting stents. The benefit of sirolimus (or the ‘limus’ group) as an anti-proliferative drug, as compared to paclitaxel, has been documented in several DES trials [57,58,59]. Its main benefits include the cytostatic mode of action (compared to the cytotoxic effect of paclitaxel) and increased anti-restenotic effect. Moreover, sirolimus, compared to paclitaxel, has a lower lipophilicity but a wider therapeutic window. For stent-based local drug delivery, sirolimus must be released for a period of several weeks to achieve effective inhibition of neointimal proliferation. Preclinical studies have demonstrated the feasibility of sirolimus balloon coating in a dose range of 1 to 7 μg per mm^2^ balloon surface, with varying amorphous or crystalline formulations [60].

It was commonly thought that only sustained drug release would ensure persistent prevention of restenosis after angioplasty and stent implantation [61]. Considering that the inhibition of neointimal proliferation by sirolimus-coated balloons (sDCBs) in the porcine model was similar to the corresponding effect of sirolimus-eluting stents, a possible clinical indication for sirolimus-coated balloons was suggested to be drug-eluting stent restenosis [60]. In 2016, the first sirolimus-coated DCB (MagicTouch) obtained the CE mark. The technology designed for this device consists of the entrapment of sirolimus in a protective lipophilic package, which allows diffusion and penetration into the arterial wall during balloon inflation, overcoming the low lipophilicity of sirolimus. The package is composed of nanospheres of 100–300 nm in diameter. The total drug dosage corresponds to 1.25 mg/mm^2^ of balloon surface area (within the therapeutic window of sirolimus). In a prospective, multicenter clinical registry, MagicTouch sDCBs showed good immediate performance and an adequate and encouraging safety profile at 12 months [6].

In 2019, a study on the treatment of coronary DES restenosis by sDCBs showed similar efficacy in terms of late lumen loss (LLL) as compared to the SeQuent Please pDCB [62].

A subsequent indirect comparison between pDCB and sDCB found no significant difference in clinical endpoints at 12-month follow-up (*p* = 0.89 for MACE) [63], and this result was then confirmed by randomized clinical trials. Recently, in fact, sDCB proved to be non-inferior to pDCB in regards to LLL, either in in-stent restenosis (lumen loss in-segment at 6 months; mean difference between sDCB and pDCB 0.01–95% CI: −0.23 to 0.24; non-inferiority at a predefined margin of 0.35 shown [64]) or in de novo lesions (lumen loss at 6 months; mean difference 0.08–95% CI: −0.07 to 0.24, although negative lumen loss was more frequent in the pDCB group (60% vs. 32% of lesions; *p* = 0.019) [16]. However, these studies did not show any difference in clinical events [16,64]. Commercially available DCBs’ characteristics are summarized in Table 1.

## 3. DCBs in De Novo Lesions: Different Anatomical Settings

### 3.1. De Novo Lesions in Small Vessels

The International DCB Consensus Group defines ‘small vessel disease’ (SVD) as a lesion in a vessel having a reference diameter (the mean diameter of the vessel proximal and distal to the lesion) of less than 3 mm [7]. SVD is quite common in clinical practice, in particular in some patient subpopulations, such as patients with diabetes [65]. Despite the technological improvement of PCI devices, percutaneous revascularization in patients with small vessel disease is still burdened by an increased rate of adverse events [66,67].

Revascularization with DES is effective in both large and small vessels, but in patients with SVD, DES implantation has shown an increased risk of late lumen loss (LLL), in-stent restenosis and other adverse clinical events [66].

In fact, the minimum lumen diameter (MLD) increases acutely after PCI and decreases at follow-up, causing the so-called LLL (difference between the post-procedural vessel diameter and the vessel diameter at follow-up), mainly due to neointimal proliferation or hyperplasia [16]. Small vessels have less ability to comply with the formation of neointimal hyperplasia since their thickness is independent of vessel diameter. Therefore, minimizing LLL is crucial in the treatment of patients with SVD to improve long-term outcomes. These pathological aspects have increased the interest around the ‘nothing left behind’ strategy in this setting.

Early data on the feasibility and safety of treating coronary lesions with DCBs in de novo SVD [17,18,19,68,69,70,71] paved the way to several randomized studies that have investigated the efficacy of DCBs in the treatment of SVD compared with plain old balloon angioplasty (POBA), bare metal stents (BMS) and DES.

The first randomized trial specifically addressing patients with SVD was the PICCOLETO Trial (Paclitaxel-Coated Balloon versus Drug-Eluting Stent During PCI of Small Coronary Vessels), which was published in 2010. In this study, 57 patients with SVD (reference diameter ≤2.75 mm) were randomized to pDCB (n = 28, Dior) or first generation DES (n = 29, paclitaxel-eluting stent Taxus). The study was stopped after 6 months due to an increase in major cardiovascular adverse events in the DCB group (36% vs. 14%, *p* = 0.054); the DCB group also had a higher rate of target vessel restenosis (32.1% vs. 10.3%, *p* = 0.043) and higher percent diameter stenosis (43.6% vs. 24.3%, *p* = 0.029) [20]. According to the authors, use of the first generation DCBs involved a lower concentration of drug at tissue level and was less effective in inhibition of neointimal proliferation. Moreover, only 25% of lesions were prepared by balloon pre-dilatation [72].

In the BELLO (Balloon Elution and Late Loss Optimization) trial, 182 patients with SVD (diameter <2.8 mm) were randomized to PCI with pDCB (n = 90, In.Pact FALCON) or paclitaxel-eluting stent (n = 92, Taxus Liberté). Stent implantation (using BMS) was required as a bailout strategy in 20% of patients in the DCB group. Pre-dilatation of target lesions was performed in 97% of cases. The rate of MACE (10% in DCB group vs. 16.3% in DES, *p* = 0.21), target lesion revascularization (TLR) (4.4% in DCB vs. 7.6% in DES, *p* = 0.37) and angiographic restenosis (8.9% in DCB vs. 14.1% in DES, *p* = 0.25) was similar in both groups after 6 months [21]. Notably, the incidence of diabetes mellitus (DM) was high in both patient groups (43.3% in DCB group and 38% in DES group). At the subgroup analysis phase, patients with DM treated with DCB had a lower LLL than patients with diabetes treated with DES (0.05 ± 0.41 mm in the DCB group vs. 0.32 ± 0.52 mm in the DES group, *p* = 0.001) [73]. Interestingly, a better long-term outcome was found for pDCB than DES at a 3-years follow up (MACE rate 14.4% in DCB group and 30.4% in DES group, *p* = 0.015) [22].

The first trial that compared DCB with second generation DES was the BASKET-SMALL 2 trial, which randomized 758 patients with SVD (diameter < 3 mm) to treatment with a pDCB (n = 382, Sequent Please) or with a second generation DES-eluting paclitaxel or everolimus (n = 376, Taxus Element or Xience). Pre-dilation was performed in all lesions and patients were excluded from the study in case of TIMI flow <3, high-grade dissection or residual stenosis >30% after balloon angioplasty. The rate of MACE, cardiovascular death, myocardial infarction and target vessel revascularization (TVR) did not differ between the two groups at 12 months [23]. Efficacy of treatment with DCB versus DES was confirmed at the 3-year follow-up [24].

In the RESTORE SVD trial, published in 2018, 230 patients (reference vessel diameter ≥2.25 mm and ≤2.75 mm) were randomized to PCI with paclitaxel coated balloon (n = 116) or zotarolimus-eluting stent (n = 114, Resolute Integrity/Medtronic). At the 9-month angiographic follow-up, patients in the DCB group had a lower MLD than with DES. Furthermore, there were no differences observed between the two groups in TLR, cardiac death, myocardial infarction and revascularization of target vessel. The rate of target lesion failure was similar in the two groups at 12 and 24 months [25,26].

In the PICCOLETO II trial (Drug eluting balloon efficacy for small coronary vessel disease treatment), published in 2020, 232 patients with SVD (2–2.75 mm) were randomized to treatment with paclitaxel-coated balloon (n = 118, Elutax) or everolimus-eluting stent (n = 114, Xience). DES-treated patients had greater in-lesion acute gain than DCB-treated patients; however, late lumen loss of DCB-treated lesions was significantly reduced as compared to those treated with DES (0.04 mm vs. 0.17 mm, *p* < 0.001 for non-inferiority), but there was no significant difference regarding MLD and percent diameter stenosis at 6 months. At a 12-month follow-up, there were no significant differences between the two groups in MACE, myocardial infarction and target vessel thrombosis [74].

A recent trial published by Yu et al. in 2021 randomized 170 patients with coronary de novo lesion to treatment with paclitaxel-coated balloon (n = 85, Sequent please) or second generation DES (zotarolimus-eluting stent, Resolute Integrity; everolimus-eluting stent, Xience Expedition or Synergy; sirolimus-eluting stents, Firehawk) [75]. At the 9-month angiographic follow-up, MLD in the DCB group was significantly increased as compared with post-intervention level (2.02 ± 0.62 mm vs. 1.83 ± 0.44 mm, *p* < 0.001), while this trend was not observed in the DES group (2.49 ± 0.76 mm vs. 2.52 ± 0.47mm, *p* = 0.705). The primary endpoint of 9-month LLL was −0.19 ± 0.49 mm with the DCB versus 0.03 ± 0.64 mm with the DES (*p* = 0.019 for non-inferiority). At the 30-day and 12-month follow-ups, there were no significant differences between the two groups in terms of MACE, myocardial infarction, TLR, target vessel thrombosis and cardiac death. The study concluded that a DCB-only strategy for de novo coronary lesion was non-inferior to DES treatment in terms of LLL and clinical outcomes [75]. However, patients included in the study had both SVD and large vessel disease (reference diameter of 2.25–4 mm), and patients with large vessel disease comprised 40.5% of the DCB group and 54% of the DES group, while a separate data analysis of the subgroup of patients with SVD is not available [75].

Randomized controlled trials of DCB-only treatment in de novo lesions of small coronary vessels are summarized in Table 2. As observed, among “special” anatomies, small vessels represent the setting with more robust data, with studies enclosing both ACS and chronic patients and large proportion of diabetic patients. Moreover, the majority of randomized trials addressed hard clinical endpoints, such as MACE and solid compactors, as DCBs were tested against routinely used DES. These results have led to significantly consider the use of DCBs in SVD, with adequate lesion preparation and selection of a new generation DCB of appropriate caliber for RVD, and particularly in selected population subgroups, such as patients with DM or HBR patients. Indeed, results from ongoing randomized trials with longer follow-up and newer devices will further reinforce current evidence.

### 3.2. De Novo Lesions in Large Vessels

The role of DCBs in the treatment of de novo lesions in large (≥3.0 mm) coronary arteries is less settled but potentially appealing, offering the advantages to avoid stent struts malapposition, especially in vessels with irregular walls, aneurismatic dilatation or in bifurcations.

In fact, feasibility of DCB-only treatment for large vessel disease was initially derived from the inclusion of this anatomical subset in observational retrospective and prospective studies with different devices (Pantera Lux, In.Pact Falcon, and mostly Sequent Pease) [19,27,70,78]. Rosenberg et al., however, further analyzed data of their cohort of patients, comparing small and large vessels outcome after propensity matching, finding similar rates of bailout stenting (7.6% and 7.1% for large and small vessels, respectively) and MACE (6.1% and 5.7% for large and small vessels at 9 months, respectively) [79]. Although in the retrospective study by Uskela et al., larger balloon size was strongly related to technical failure (OR of 1.94) [28]. More recent studies showed favorable results of the DCB-only strategy in large vessels. A Chinese study, for example, showed a bailout stenting rate of 0.5% and absence of MACE at an average of 10.1 months of clinical follow-up in large vessel disease (vs. 1.4% MACE rate in the small vessels group, non-significant difference) [29]. Moreover, a retrospective analysis showed a good performance of DCB in comparison with DES in a population of consecutive patients affected by stable angina mostly caused by large vessel disease, with similar all-cause mortality in the entire population and after propensity matching [30]. Recently, a prospective observational study showed a low rate (4.2% at a 2-years median follow-up) of target lesion failure in vessels ≥2.75 mm, including bifurcation lesions in 45% of patients [80].

Interesting data on DCB treatment of large vessels also comes from some small randomized clinical trials (RCTs) that are summarized in Table 2. In the DEBUT trial, DCB-only treatment resulted in a lower rate of MACE compared to bare metal stents (BMS) in a high bleeding risk population, and this result was even more relevant in the large vessel subgroup, accounting for about 2/3 of the overall population. However, it should be noted that randomization was performed in this trial after successful preparation of the lesion [31]. According to MACE, treatment with DCB was non-inferior to the treatment with stents in two more RCTs: one focused on NSTEMI patients, grouping BMS and DES with an average diameter of 3.03 mm as comparator [32], and the other involving consecutive patients achieving ideal results after pre-dilation, with second generation DES as comparator (of note, nearly half the lesions were located in ≥3.0 mm vessels) [75]. These recent RCTs seem to reinforce previous reports about effectiveness of DCB in the treatment of large vessel disease, although carefulness is required in drawing definitive conclusions, considering their small dimension and heterogeneous inclusion criteria.

### 3.3. De Novo Lesions Involving Bifurcations

Coronary lesions involving bifurcations of major epicardial vessels account for 20% of coronary lesions undergoing PCI [81], but these cases present a greater technical difficulty and worse long-term outcomes than lesions that do not involve bifurcations [82].

The European bifurcation Club recommends ‘provisional stenting’ as the preferred strategy, i.e., a main vessel (MV)-only stenting in most cases and side branch (SB) stenting in cases of recoil or severe flow compromise after main branch stenting (with T stenting, T-and-protruding (TAP) or culotte technique) [83]. The upfront two-stent strategy is reserved for cases in which the ostia and the first tract of the side branch present severe and long disease and there is a large area of myocardial at risk (e.g., left main trunk (LMT) bifurcation) [83]. However, a two-stent strategy presents an increased risk of long-term mortality compared to the provisional technique [84].

Due to the increasing complexity of bifurcation lesions and growing evidence on the efficacy and safety of using DCBs in de novo lesions of small and large vessels [23,30,85], the concept of ‘nothing left behind’ with the use of DCBs in bifurcation PCI is recently gaining ground.

Several studies have been conducted on the use of DCBs in the setting of bifurcation lesions. However, most of the available data derive from registries or non-randomized trials, and their results are limited by heterogeneous bifurcation classification, PCI technique and the timing of use of DCBs. Moreover, in many of these studies, disease of the bifurcation of the LMT represented an exclusion criterion. Furthermore, in some cases, the use of DCBs in the side branch was followed by stenting of the main vessel with BMS, which is not the current best standard of care [15,86,87].

In the first randomized trial designed to investigate the efficacy of DCBs in bifurcation lesions, Stella et al. randomized 117 patients with coronary bifurcation lesions to treatment with (A) pDCB in both, MV and SB, and BMS in MB, (B) BMS in MV and regular balloon angioplasty in SB or (C) paclitaxel DES in MV and regular balloon in SB. According to the authors, pre-treatment of both MV and SB with DCB failed to show angiographic and clinical superiority over conventional BMS, and DES showed superior angiographic results than DCB and BMS [76]. In the randomized trial BABILON, which enrolled 108 patients, after dilatation of the lesion with DCB in MV and in SB in both groups, a provisional technique was performed by stenting with BMS in the pDCB group and with everolimus DES in the DES group. The group treated with BMS in the MB showed an increased incidence of MACE compared to everolimus DES [33]. However, it should be emphasized that both of these trials used an outdated strategy in the treatment of MB in control group, i.e., the use of BMS.

The ‘DCB-only’ strategy was investigated in the randomized trial PEPCAD-BIF, in which 64 patients were randomized to PCI in both MB and SB with plain old balloon angioplasty (POBA) (n = 32) or pDCB (SeQuent Please). Patients with acute coronary syndrome, heart failure, lesions involving the left main or lesions involving the proximal MV (Medina 1, x, x) were excluded from the study. At the 9-month follow-up, the LLL (the primary endpoint) was 0.13 mm in the DCB and 0.51 mm in the control POBA group [*p* = 0.013; 95 % CI (−0.66 to −0.08)]. The binary restenosis rate was 6% in the DCB group and 26% in the control group (*p* = 0.045). There were no significant differences in terms of MACE and TLF between the two groups [34]. However, the use of POBA alone in the control group in both MV and SB represents the major limitation of the study.

The feasibility and safety of a stenting approach with DES in the main vessel and DCB in the side branch was investigated in three observational studies [35,36,37]. Overall, despite some limitations (e.g., exclusion of bifurcations in proximal vessels, the absence of a control group, and the small number of patients enrolled), these three trials showed a good procedural success rate and low LLL in the side branch. The 12-month MACE rate was 5.9% in the BIOLUX trial and 19% in the SARPEDON trial [35,37]. Only one patient (2%) had myocardial infarction and there were no cardiac deaths at six-months follow up in the DEBSIDE trial [36].

In the BEYOND randomized trial, 222 patients with bifurcation lesions (excluding patients with LMT bifurcation involvement) were randomized to provisional treatment strategy with DES in the MV in both groups, followed by POBA (n = 109) or DCB (n = 113) in the SB [88]. This study demonstrated that in de novo non-LMT coronary artery bifurcations treated with provisional T-stenting, SB dilation with DCB group demonstrated better angiographic results than treatment with regular POBA at the 9-month follow-up. In fact, target lesion stenosis in the DCB group was 28.7% ± 18.7% and in the POBA group, it was 40.0% ± 19.0% (*p* < 0.0001). The LLL was also significantly lower in the DCB group than in the POBA group (−0.06 ± 0.32 vs. 0.18 ± 0.34 mm, *p* < 0.0001) [77]. On the other side, there were no significant differences between DCB and POBA in MACE or non-fatal myocardial infarctions between the groups [77]. Randomized controlled trials of DCB-only treatment in de novo lesions of bifurcations are summarized in Table 2. Of note, unprotected left main involvement was an exclusion criterion in all randomized trials, except PEPCAD-BIF, and in the BEYOND trial, other lesion locations were listed among exclusion criteria (aorto-ostial lesions, target lesion within 5 mm of the origin of the left anterior descending, left circumflex or right coronary artery) [33,34,35,76]. In summary, the available literature regarding the use of DCBs for coronary bifurcation lesions, although growing, is still limited.

In conclusion, as suggested in the Third Report of the international DCB Consensus Group, to simplify revascularization of bifurcation lesions, a DCB-only strategy may be attempted, while a DES in MB and DCB in SB strategy may be chosen in the case of compromised results during the pre-dilation stage [7]. However, while waiting for future studies, the treatment of bifurcation lesions with DCB should be evaluated based on patient characteristics and anatomy of the lesion in a case-by-case fashion.

## 4. DCBs in Specific Clinical Settings

### 4.1. Diabetes Mellitus

Diabetic patients represent about one-third of the patients undergoing PCI, although being the proportion with poorer procedural results and worst long-term outcomes.

More complex coronary anatomy, multivessel and diffuse disease, but also clinical factors, including enhanced thrombotic risk and comorbidities, have accounted for this prognostic discrepancy [88,89]. In fact, smaller vessel diameter, calcifications conditioning potential struts malapposition and under expansion, longer lesions requiring more extensive stenting, and the pro-inflammatory milieu induced by hyperglycemia have been shown to expose the patients to an increased risk of target lesion failure (TLF) and instant restenosis [90]. Despite the fact that newer-generation DES have provided clear benefits over first-generation stents, even among patients with diabetes [91], higher rates of intimal hyperplasia and lumen loss have still been described among these patients, thus representing an ideal setting for the use of DCBs. However, few data have been reported so far in the diabetic population.

In the Drug-Eluting Balloon for In-Stent Restenosis (DARE) trial, paclitaxel-eluting balloons were compared with the everolimus-eluting stent (Xience) in the treatment of any ISR. In patients with ISR and DM, the paclitaxel-eluting balloon resulted in similar 6-months in-segment minimal lumen diameter and comparable rates of major adverse events compared to Xience, and in-segment late loss at 6 months was significantly lower in the paclitaxel-eluting balloon arm [92].

A Bayesian meta-analysis by Lee et al. comparing different revascularization strategies for ISR in diabetics showed that local drug delivery by DEB or DES was markedly better than POBA in preventing TLR; however, treatment with DEB showed a trend of less development of MI than did treatment with DES [93].

Moreover, in de novo lesions, the use of DCB could present potential advantages, allowing to avoid or reduce stent length and then to further enhance the pro-inflammatory response induced by the permanent metallic material in the coronary artery.

In a sub analysis of the Basel Stent Kosten Effektivitäts Trial Drug Eluting Balloons vs. Drug Eluting Stents in Small Vessel Interventions [BASKET-SMALL 2] trial [94], the 252 patients with DM displayed similar outcomes with DCB as compared to DES, although the rate of TVR was significantly reduced with DCB. A similar favorable conclusion was reached in a recent meta-analysis by Megaly et al. [95] including three studies ( with 440 de novo lesions) and in a Chinese registry [96]. However, despite these promising data, the large scale use of DCBs in patients with DM still requires to be confirmed in RCTs dedicated to this specific subset of patients.

### 4.2. High-Bleeding Risk Patients

The increasing complexity of patients undergoing PCI has led to the treatment of elderly and frailer patients being exposed to an enhanced risk of bleeding events on DAPT [97].

Although the duration of DAPT has been progressively shortened with newer generations of DES, increasing evidence has emerged of the lower thrombotic risk after DCBs than with DES.

In a recent International Consensus Report on Drug-Coated Balloons, the recommended duration of DAPT was 4 weeks after a DCB-only strategy in de novo vessels and in patients with chronic coronary syndrome [7]. Nevertheless, according to the 2018 European Society of Cardiology/European Association for Cardio-Thoracic Surgery Guidelines on myocardial revascularization, DAPT should be considered for 6 months in patients with chronic coronary syndrome treated with DCB [98].

Moreover, in a large all-comers single center registry, among the 487 PCI procedures performed with DCBs, the median duration of DAPT was 1 month, while 4% of the procedures were performed on single-antiplatelet therapy in the case of extremely high bleeding risk, suggesting the feasibility of such a strategy with a low rate of thrombotic complications. In fact, acute vessel closure occurred only in one case (0.2%) after DCB treatment [28]. Similarly, in a summary of randomized clinical trials and registries, no cases of an acute or a subacute thrombosis were reported after a DCB-only strategy in around 1500 PCI, including stable and ACS patients [99].

While data from trials specific for HBR patients are still lacking, it should be noted that a sub analysis of the BASKET-SMALL 2 trial compared DCB to DES in high bleeding risk (HBR) patients (24% in DES group and 17% in DCB group). It was shown that although HBR patients were three times more at risk of MACE compared to non-HBR patients, within the HBR group, there were no significant differences in primary endpoints in patients treated with DCB or DES [100].

Of note, the Asia-Pacific Consensus Group recently recommended that patients should continue the second antiplatelet agent for at least 1–3 months after PCI with DCB for ISR, while for the treatment of de novo coronary disease (except ACS with DCB only), patients should receive DAPT for at least 1 month [101].

### 4.3. Acute Coronary Syndrome

Patients with ACS represent a population with a particularly enhanced risk of thrombosis and PCI failure. Diffuse vessel spasm and the presence of thrombotic material have been associated to distal embolization and microvascular obstruction, promoting the no-reflow phenomenon and more frequent vessel under sizing and struts malapposition [102].

Therefore, deferred stenting has been shown to offer potential advantages, especially in patients with STEMI, as compared to immediate PCI with stent.

In this context, the use of DCBs could offer the advantages of restoring coronary flow and promote vascular healing without the risks connected to inadequate stent sizing.

In fact, deferred stenting is emerging over immediate stent deployment for the management of primary PCI lesions, especially in settings with high thrombus burden, preventing the no-reflow phenomenon and stent under sizing [103].

The initial pilot study paclitaxel-eluting balloon angioplasty in the Primary Percutaneous coronary intervention in Amsterdam (PAPPA, [38]) enrolled one hundred patients presenting with ST-elevation MI, of who 59 were treated with DCB alone. They showed an extremely low rate of MACE (%) with such a strategy, although this study did include a quite selected population of young patients with soft non-calcified and not tight stenoses and without diabetes. In effect, a similar conclusion was reached by Verdoia et al. in an all-comers registry showing that complex and type C lesions were independent predictors of adverse cardiovascular events (adjusted OR [95% CI] = 1.78 [1.05–2.95], *p* = 0.03), with no impact on survival [104].

In the REVELATION (Drug-Coated Balloon Versus Drug-Eluting Stent in Acute Myocardial Infarction) trial for ST-segment elevation myocardial infarction, no outcome difference was observed between the DCB and DES groups, even at two-years follow-up [39].

Similar results were reported in the PEPCAD NSTEMI trial [32] and in two different subgroup analyses, each including over 200 ACS patients with de novo lesions: the BASKET-SMALL 2 trial and the Finnish registry by Uskela et al. [28,105]. In the latter, as expected, the total mortality and rate of MACE were higher in ACS patients than in stable CAD (mortality: 9.3% in ACS vs. 2.3% in stable CAD; MACE: 12% in ACS vs. 7.1% in stable CAD). However, the rate of ischemia-driven target lesion revascularization was low in both stable and ACS patients (1.4% and 2.8%, respectively), with extremely low rates of acute vessel closure (0.2%) and bailout stenting (12%) [28].

In the recent DEB-AMI trial [106], among the 40 patients treated in the context of STEMI major angiographic and safety endpoints, comparable results were observed between paclitaxel-eluting stents and balloons, with no acute or late thrombotic events in the DCB-only group. However, the long term lumen loss was superior with DCB as compared to DES (0.5160.59 mm vs. 0.2160.32 mm, respectively; *p* < 0.01). Similarly, Caiazzo et al. showed in the SELFIE prospective registry a good safety and efficacy profile for the treatment of de novo coronary lesion and ISR also with a sirolimus-coated DCB, although the study was not powered for evaluating outcome endpoints [107]. However, more solid data have recently come also from a large meta-analysis [108].

In conclusion, while waiting for large scale trials dedicated to ACS patients, caution is necessary when using DCBs in this setting.

## 5. Conclusions

**Figure 1:**
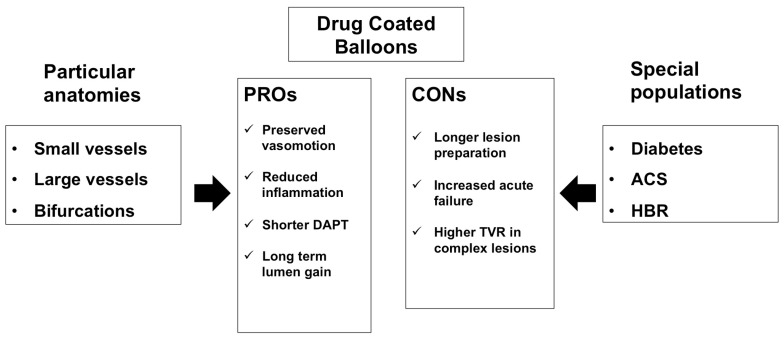
Central figure.

Scientific interest is growing with regard to a DCB-only approach in de novo lesions. Available data support this strategy mostly in SVD, where it could be a valid alternative treatment to DES after optimal balloon angioplasty. However, recent studies have focused on other anatomical (large vessels, bifurcations) or clinical (diabetes mellitus, HBR patients, ACS) settings, providing promising results that should be confirmed in properly designed randomized clinical trials (Figure 1: central figure). Indeed, a rigorous patients’ selection and adequate preparation of the target lesion represent mandatory indications in the treatment of de novo lesions in order to optimize results, prevent bailout stent implantation and secure the long-term maintenance of the results.

## Figures and Tables

**Table 1:** Characteristics of commercially available DCBs.

Company	Product	Drug	Drug Concentration (μg/mm^2^)	Excipient	Balloon Diameter (mm)	Balloon Length (mm)	De Novo Lesions Assessment
B. Braun Melsungen AG (Melsungen, Germany)	SeQuent Please	Paclitaxel	3	Iopromide (PACCOCATH technology)	2–4	10–40	RCT: Basket small 2 (n = 382) [23,24]; DEBUT (n = 103) [31]; PEPCAD NSTEMI, (n = 104) [32]; Funatsu et al. (n = 92) [40]; Gobic et al. (n = 38) [41]; Shin et al. (n = 20) [42]; PEPCAD-BIF (n = 32) [34]; BABILON (n = 52) [33]; Nishiyama et al. (n = 30) [43]. OS: Rosenberg et al. (n = 731) [19]; Uskela et al. (n = 463) [28]; Yu et al. (n = 595) [29]; Venetsanos et al. (n = 4483) [44]; Onishi et al. (n = 196) [45]; Merinopoulos et al. (n = 408) [30]; Funayama et al. (n = 111) [46].
Medtronic-Invatec (Roncadelle, Italy)	PREVAIL (previously In.Pact Falcon)	Paclitaxel	3.5	Urea	2–4	10–30	RCT: BELLO (n = 90) [21,22]. OS: Venetsanos et al. (n = 1071) [44]; Merinopoulos et al. (n = 21) [30]; FALCON Registry (n = 757) [47]; PREVAIL (n = 50) [48].
Eurocor GmbH (Bonn, Germany)	Dior I	Paclitaxel	3	No Excipient (Nanoporous balloon surface)	2–4	10–30	RCT: PICCOLETO SMALL VESSELS (n = 57) [20]. OS: 001-DIOR (n = 49) [49]; Valentines II (n = 103) [17]; DEAR (n = 91) [50]; Vaquerizo et al. (n = 104) [18].
	Dior II			Shellac			
Biosensors International (Singapore)	Biostream	Paclitaxel	3	Shellac	2–4	15–30	-
Biotronik AG (Baar, Switzerland)	Pantera Lux	Paclitaxel	3	BTHC	2–4	10–30	RCT: REVELATION (n = 60) [39]; DELUX large (n = 105) [27]. OS: Venetsanos et al. (n = 1161) [44]; PAPPA (n = 100) [38]; BIOLUX-I (n = 35) [35]; SARPEDON (n = 58) [37].
Angioscore/Philips (Fremont, California)	AngiosculptX	Paclitaxel	3	NDGA	2–3.5	10–20	-
Boston Scientific (Natick, Massachusetts)	Agent	Paclitaxel	2	ATBC	2–4	12–30	-
Aachen Resonance GmbH (Aachen, Germany)	Elutax I-Elutax II	Paclitaxel	2.2	No excipient	2–4	10–40	RCT: PICCOLETO (n = 118) [20]. OS: DCB-RISE (n = 544) [51].
	Elutax SV			Dextran Sulfate			
Blue Medical BV/Wellinq (Helmond, The Netherlands)	Protégé/Protégé NC	Paclitaxel	3	BTHC	2–4	10–30	OS: PEARL Registry (n = 131) [52].
Minvasys (Gennevilliers, France)	Danubio	Paclitaxel	2.5	BTHC	1.5–4	10–40	OS: DEBSIDE (n = 52) [36].
iVascular (Barcelona, Spain)	Essential	Paclitaxel	3	BTHC	1.5–4.5	10–40	OS: Abellas-Sequeiros et al. (n = 71) [53].
Cardionovum (Bonn, Germany)	Restore	Paclitaxel	3	Shelloic Acid	2–4	15–30	RCT: RESTORE SVD (n = 116) [25,26].
Eucatech (Weil am Rhein, Germany)	Support C	Paclitaxel	3	BTHC	2–4	10–30	-
Nano Therapeutics (Surat, India)	Curex PTCA	Paclitaxel	2.3	BTHC	2–4.5	9–41	-
B.Braun Melsungen AG (Melsungen, Germany)	SeQuent SCB	Sirolimus (crystalline)	4	-	2.5–3.5	15–30	OS: Ahmad et al. (n = 70) [16].
Concept Medical Inc. (Tampa, Florida)	Magic Touch	Sirolimus	1.27	Phospholipid Based Excipient	1.5–4	10–40	RCT: TRANSFORM I (n = 114) [54]. OS: EASTBOURNE (n = 596) [6]; NANOLUTE (n = 225) [55]; FASICO (n = 18) [56].
Med Alliance (Nyon, Switzerland)	Selution	Sirolimus	1	MicroReservoirs embedded with CAT coating, proprietary amphipathic lipid technology	2–7	20–150	-
Orchestra Biomed /Terumo (New Hope, Pennsylvania)	Virtue SAB	SirolimusEFR Formulation	-	No Excipient (Perforated Balloon Surface: angioinfusion)			-

ATBC: Acetyl tributyl citrate. BTHC: Butyryl-tri-hexyl citrate. CAT: Cell Adherent Technology. NDGA: nordihydroguaiaretic acid. OS: Other (i.e., non-randomized) studies. RCT: Randomized clinical trials.

**Table 2:** Randomized controlled trials of DCB-only treatment in de novo lesions of small coronary vessels, large coronary vessels and bifurcations. pDCB: paclitaxel-coated drug-coated balloon. DES: drug-eluting stents. LLL: late lumen loss. MACE: main adverse cardiovascular events. TLF: target lesion failure.

Study	N	DCB	Comparator	Follow Up	Main Findings—Angio	Main Findings—Clinical	Diabetes Mellitus	ACS	Female Gender
Small vessels									
PICCOLETO [20]	57	Dior pDCB	Taxus Liberté DES	6 months	higher rate of target vessel restenosis; higher percent diameter stenosis	MACE: higher rate	37.9% (in DCB group)	46.4% (unstable angina in DCB group)	31.4% (in DCB group)
BELLO [21,22]	182	IN.PACT Falcon pDCB	Taxus Liberté DES	6 months angio, 12 months clinical, 3 years clinical	lower LLL	MACE at 1 year: similar; MACE at 3 years: lower	40.7%	-	28.3%
BASKET-SMALL 2 [23,24]	758	Sequent Please pDCB	Taxus Element DES and Xience DES	6 months angio, 12 months clinical, 3 years clinical	LLL: no difference	MACE: non inferiority. No significant differences in cardiac death, stent thrombosis and major bleeding at 1- and 3-year follow-ups	31.9% (in DCB group)	31.8%	23% (in DCB group)
RESTORE-SVD [25,26]	230	Restore pDCB	Resolute Integrity DES	9 months angio, 12 months clinical, 2 years clinical	percentage stenosis: non inferiority	No significant differences in TLF	46% (in DCB group)	8%	33% (in DCB group)
PICCOLETO II [74]	232	Elutax pDCB	Xience DES	6 months angio, 12 months clinical	lower LLL; percent diameter stenosis and minimal lumen diameter not significantly different	No significant differences in MACE	65.6% (in DCB group)	44.2% (in DCB group)	33.1% (in DCB group)
Large vessels									
Yu et al. [75]	183 (58.4% > 3 mm)	Any DCB	Any DES	9 months angio	lumen loss (LLL) of target lesions at angiographic follow-up	No significant differences in LLL and 12-months MACE	19%	0%	26.2%
DEBUT [31]	208 (58.4% > 3 mm)	Sequent Please pDCB	Omega BMS	9 months	MACE	Lower rate of MACE with DCB vs. BMS	26% (in DCB group)	46%	37%
PEPCAD NSTEMI [32]	210	Sequent Please pDCB	EES or BMS	9 months	TLF	Non-inferiority of DCB	26.9% (in DCB group)	100%	33.7% (in DCB group)
Bifurcations									
Stella et al. [76]	117	DIOR-I pDCB	LibertèBMS or DES	12 months	LLL	DCB not superior to MB conventional stenting	5%	-	37.5% (in DCB group)
BABILON [33]	108	Sequent Please pDCB	Xience DES	9 months	LLL	Higher LLL with DCB + BMS vs. DES	26.9% (in DCB group	44.2% (in DCB group	36.5% (in DCB group
PEPCAD BIF [34]	64	Sequent Please pDCB	POBA	9 months	LLL	Superiority of DCB vs. POBA	35.9%	23.4%	36.6%
BEYOND [77]	222	Bingo pDCB	POBA	9 months	Target lesion stenosis	Superiority of DCB vs. POBA	30.1% (in DCB group)	92% (in DCB group)	20.3% (in DCB group)

## Data Availability

No new data were created or analyzed in this study. Data sharing is not applicable to this article.
